# Bioinformation and Monitoring Technology for Environmental DNA Analysis: A Review

**DOI:** 10.3390/bios15080494

**Published:** 2025-08-01

**Authors:** Hyo Jik Yoon, Joo Hyeong Seo, Seung Hoon Shin, Mohamed A. A. Abdelhamid, Seung Pil Pack

**Affiliations:** 1 Institute of Natural Science, Korea University, Sejong-ro 2511, Sejong 30019, Republic of Korea; hyojik88@korea.ac.kr; 2 Department of Biotechnology and Bioinformatics, Korea University, Sejong-ro 2511, Sejong 30019, Republic of Korea; sjh0413@korea.ac.kr (J.H.S.); bimilbunho@korea.ac.kr (S.H.S.); mabdelhamid@su.edu.om (M.A.A.A.); 3 Biology Department, Faculty of Education and Arts, Sohar University, Sohar 311, Oman

**Keywords:** bioinformatics, environmental genomics, environmental monitoring

## Abstract

Environmental DNA (eDNA) analysis has emerged as a transformative tool in environmental monitoring, enabling non-invasive detection of species and microbial communities across diverse ecosystems. This study systematically reviews the role of bioinformation technology in eDNA analysis, focusing on methodologies and applications across air, soil, groundwater, sediment, and aquatic environments. Advances in molecular biology, high-throughput sequencing, bioinformatics tools, and field-deployable detection systems have significantly improved eDNA detection sensitivity, allowing for early identification of invasive species, monitoring ecosystem health, and tracking pollutant degradation processes. Airborne eDNA monitoring has demonstrated potential for assessing microbial shifts due to air pollution and tracking pathogen transmission. In terrestrial environments, eDNA facilitates soil and groundwater pollution assessments and enhances understanding of biodegradation processes. In aquatic ecosystems, eDNA serves as a powerful tool for biodiversity assessment, invasive species monitoring, and wastewater-based epidemiology. Despite its growing applicability, challenges remain, including DNA degradation, contamination risks, and standardization of sampling protocols. Future research should focus on integrating eDNA data with remote sensing, machine learning, and ecological modeling to enhance predictive environmental monitoring frameworks. As technological advancements continue, eDNA-based approaches are poised to revolutionize environmental assessment, conservation strategies, and public health surveillance.

## 1. Introduction

### 1.1. Importance of Environmental Monitoring

Environmental monitoring is a cornerstone for understanding current ecological conditions and serves as a predictive tool for anticipating future challenges [1]. Rapid urbanization has led to phenomena such as urban heat islands and accelerated deforestation, emphasizing the urgent need for effective monitoring [2]. Advances in remote sensing, the Internet of Things (IoT), and automated sensor networks are increasingly integrated into monitoring frameworks, providing continuous, high-resolution datasets that enhance our ability to detect subtle changes across vast spatial and temporal scales [3]. For example, real-time data from satellite imagery and ground-based sensors can be cross-referenced with in situ measurements to validate observations [3]. Environmental monitoring is now a key component of adaptive management: by continuously updating datasets, scientists and policymakers can rapidly adjust strategies in response to new insights or unexpected disturbances [4]. The integration of citizen science further enriches these datasets by democratizing data collection and increasing community engagement in environmental stewardship [5]. This collaborative approach expands geographic coverage and raises public awareness, fostering a more proactive response to degradation and climate change [6]. The growing recognition of ecosystem services, which are the benefits that natural systems provide to humanity, has amplified the need for robust monitoring programs [7]. Quantitative assessments of services such as water purification, carbon sequestration, and soil fertility are vital for informed decisions on land use, resource allocation, and conservation priorities. Thus, environmental monitoring serves a dual purpose: safeguarding ecological integrity and ensuring sustainable development. An ideal monitoring technique is widely deployable, minimally invasive, and yields robust and reproducible data, qualities that environmental DNA (eDNA) uniquely offers [8,9,10].

### 1.2. Importance of eDNA in Environmental Monitoring

eDNA refers to genetic material shed by organisms through processes such as excretion, secretion, and the shedding of skin or tissues [11,12]. It can be extracted directly from environmental samples such as water, soil, sediment, or air without first isolating any target organisms. Advances in molecular biology, high-throughput DNA sequencing, and bioinformatics over the past two decades have enabled efficient collection, amplification and analysis of eDNA from diverse habitats [13]. One major advantage over traditional biomonitoring methods is high-sensitivity. Even organisms present at low abundance or those that are difficult to trap can be detected via their genetic signature, enabling early identification of invasive or cryptic species and offering a cost-effective alternative to labor-intensive methods such as electrofishing, diver surveys, and baited net trapping. Another key benefit is non-invasive sampling; for example, filtering a few liters of water causes minimal disturbance compared with nets or electrofishing, and sampling soil cores preserves habitat integrity [14,15]. Recent advances have moved beyond basic analytics with the development of denoising pipelines (e.g., DADA2) that correct sequencing errors; machine-learning classifiers (e.g., Random Forests) that enable more accurate taxonomic assignments; and big-data platforms (e.g., Hadoop) capable of handling longitudinal, multi-site eDNA datasets [16,17,18]. These tools reduce false positives, improve rare-species detection limits, and allow high-frequency sampling data to be integrated into real-time monitoring dashboards. By collecting and sequencing DNA from environmental samples, researchers can obtain a snapshot of biodiversity from bacteria and fungi to plants and animals all at once. This approach, often referred to as metabarcoding, allows for more holistic assessments of ecosystem health and provides insights into the presence of pathogens or indicator species. To extract deeper insights, recent studies combine eDNA metabarcoding with metagenomic sequencing, enabling functional gene expression analysis and pollutant (microbiome interaction) studies at ecosystem scales. Despite their potential, eDNA-based approaches still face challenges. DNA in the environment can degrade quickly under heat, UV light, or microbial activity, limiting the temporal window for species detection. Contamination risks can arise at every step, from field sampling to laboratory analyses. Additionally, interpretation of eDNA data can be complex: detecting DNA does not necessarily mean that the organism is alive or reproducing in that environment. Nonetheless, standardized protocols, stringent quality assurance measures, and continuous improvements in sequencing technologies are steadily addressing these limitations.

A critical consideration in environmental DNA analysis is the risk of false-positive and false-negative detections. False positives typically result from contamination during sample handling or transport of DNA from non-target locations, potentially leading to incorrect conclusions about species presence or distribution. False negatives, on the other hand, often occur due to low target DNA abundance, rapid DNA degradation, inefficient extraction processes, or analytical sensitivity limitations, resulting in missed detections of rare or elusive organisms. To address these challenges, stringent contamination control protocols, including the use of negative controls, sterile equipment, and the spatial separation of sample processing steps, are essential. Additionally, optimizing DNA extraction methods and employing sensitive detection techniques such as digital polymerase chain reaction (dPCR) or high-throughput sequencing can enhance the reliability of detections.

### 1.3. Trend of eDNA Monitoring

**Figure 1:**
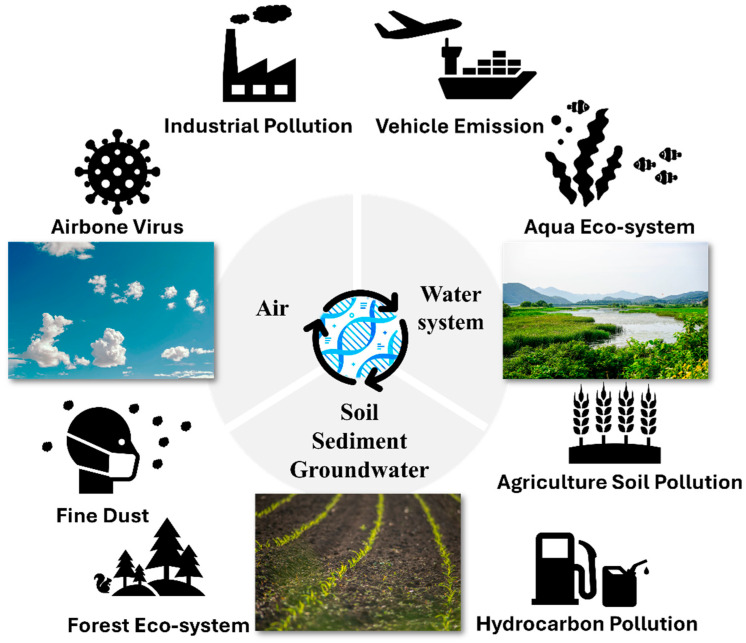
eDNA as a tool for environmental assessment.

Since eDNA was first popularized as a tool for aquatic species detection in the mid-2000s, the field has expanded rapidly in scope and sophistication [19]. Early applications predominantly focused on fisheries and amphibian conservation, using eDNA to detect low-density or rare species. Over time, improvements in next-generation sequencing (NGS) platforms and bioinformatic pipelines enabled more comprehensive biodiversity surveys via metabarcoding, ushering in an era of multi-species detection across diverse ecological niches [20,21,22]. Beyond water, airborne eDNA captures tracks of pollen, fungal spores, and pathogen dispersal; soil eDNA surveys now inform nutrient-cycling and contaminant-degradation studies (Figure 1). Coupled with time-series machine-learning models [23], these methods support near-real-time ecosystem forecasting and early warning of ecological disturbances. Researchers are exploring airborne eDNA to track the spread of pathogens, pollen, or fungal spores, while studies on soil microbial communities provide insights into nutrient cycling, pollution degradation, and ecosystem functioning [11]. In parallel, eDNA sampling in industrial contexts has proven valuable for real-time monitoring of pathogens and antibiotic resistance genes, with significant implications for public health. In addition to spatial expansion, the temporal resolution of eDNA monitoring is improving. Remote or in situ sequencing platforms, as well as miniaturized eDNA sampling devices, are being tested to capture continuous or near-real-time data [24]. Coupled with machine learning models and advanced bioinformatics, these innovations hold promise for developing early-warning systems in environmental monitoring [25,26]. Policymakers, environmental agencies, and conservation organizations are increasingly recognizing the strategic value of eDNA, leading to discussions about formalizing eDNA-based methods into regulatory frameworks. Standardization consortia (e.g., ISO TC 276 WG7) are drafting guidelines for eDNA assay validation, while pilot programs in the EU and US EPA are evaluating eDNA metrics for water quality criteria. As multidisciplinary collaborations grow, standards and guidelines will continue to improve. The synthesis of eDNA data with other monitoring tools (e.g., remote sensing and satellite imaging) and the integration of big-data analytics will further enhance our capacity to detect, understand, and respond to environmental change. In doing so, eDNA stands poised to revolutionize how we observe and protect life on Earth.

### 1.4. Technologies in eDNA Monitoring

The advancement of environmental DNA monitoring has been driven by breakthroughs in molecular biology, sequencing technologies, bioinformatics, and sample collection methods. Improvements in DNA extraction and preservation techniques have enhanced the recovery of fragmented DNA from various environmental matrices such as water, soil, sediment, and air. PCR innovations (from endpoint PCR to quantitative PCR [27] and droplet digital PCR [28]) now achieve detection limits as low as one copy per microliter for qPCR and down to 0.13 copies per microliter for ddPCR, enabling quantification of rare targets and absolute abundance estimates without requiring standard curves. The rise of high-throughput sequencing (HTS), particularly NGS and metabarcoding, has enabled large-scale biodiversity assessments by analyzing mixed eDNA samples. Additionally, advances in bioinformatics and artificial intelligence (AI) have improved species identification through expanding reference databases, machine learning algorithms, and big data analytics. Deep-learning models (e.g., convolutional autoencoders) are being trained on synthetic mock communities to denoise ultra-short reads (<100 bp) and infer haplotype variants, expanding reference databases for dark taxa [29]. Innovations in eDNA collection methods, such as improved filtration systems, passive samplers, and airborne eDNA technology, have expanded monitoring capabilities [30]. Moreover, the development of field-deployable technologies, including portable PCR and real-time sequencing, has made on-site eDNA analysis more accessible. Integration with remote sensing, geographic information systems (GIS) mapping, and ecological modeling has further strengthened eDNA’s role in environmental monitoring and conservation efforts. Emerging lab-on-a-chip platforms integrate isothermal amplification methods such as loop-mediated isothermal amplification (LAMP), which rapidly amplifies DNA at a constant temperature, and recombinase polymerase amplification (RPA), a low-temperature, rapid amplification technique, with clustered regularly interspaced short palindromic repeats and CRISPR-associated protein (CRISPR-Cas) sensors, which enable highly specific nucleic acid recognition and cleavage. These systems are often coupled with smartphone-controlled fluorescence readers, enabling on-site, multi-target pathogen and biodiversity detection within 30 min [31,32]. These advancements have transformed eDNA from an emerging research tool into a powerful, scalable method for biodiversity conservation, environmental impact assessments, and climate change studies.

### 1.5. Literature Search Strategy

Searches were conducted in Google Scholar, Web of Science, and Scopus for articles published from 1995 to 2025. A total of 160 reviewed articles were included for analysis (28 published between 1995 and 2015 and 117 published between 2016 and 2025). The final reference list includes an additional 15 methodological guidelines and background sources, bringing the total to 160 references. Titles and abstracts were screened independently by two authors (H.Y. and J.S.). Included studies met the following criteria: peer-reviewed English-language publications reporting original data on eDNA methods or applications in aquatic, terrestrial, or aerial environments, while conference abstracts, book chapters, non-English publications, and studies unrelated to environmental DNA monitoring were excluded. Any disagreements during the screening were discussed until a full agreement was reached or, if necessary, resolved by consultation with other authors (S.S., M.A., and S.P.).

## 2. Technology and Applications of eDNA Monitoring

eDNA analysis is proving to be a transformative technology for monitoring pollution across a range of environmental compartments. From air to soil, water, and solid waste, eDNA offers sensitive, non-invasive, and scalable insights into how contaminants, microbial communities, and organisms interact within various ecosystems. This chapter provides an overview of how eDNA is applied in three key domains of environmental pollution monitoring.

### 2.1. Methodologies and Environmental Applications of Airborne eDNA

#### 2.1.1. Rationale for Airborne eDNA Analysis

Air pollution poses significant threats to biodiversity and human health, traditionally monitored through metrics such as particulate matter (PM) [33,34,35], ozone [36,37], sulfur dioxide [38,39], and nitrogen oxides [39,40]. However, these conventional air quality monitoring techniques primarily focus on chemical pollutants, overlooking the biological impact of air pollution on ecosystems. Air pollution also carries biological components, including microorganisms [39,41], spores [42], pollen [43], and various volatile organic compounds [44]. eDNA analysis offers a novel approach by enabling the detection of a broad range of organisms, from microorganisms to plants and animals, directly from air samples. The concept of airborne eDNA monitoring builds on the idea that genetic material can attach to fine particulates [45,46] or exist in aerosols [46,47]. By capturing and analyzing this genetic material, researchers can assess microbial diversity, pathogen presence, and even the distribution of allergenic species in urban, industrial, or natural areas. This technique provides a more comprehensive understanding of the environmental impact of air pollution, allowing for the assessment of the biological diversity and health of ecosystems in polluted areas without invasive sampling.

#### 2.1.2. Sampling Methods and Protocols

**Figure 2:**
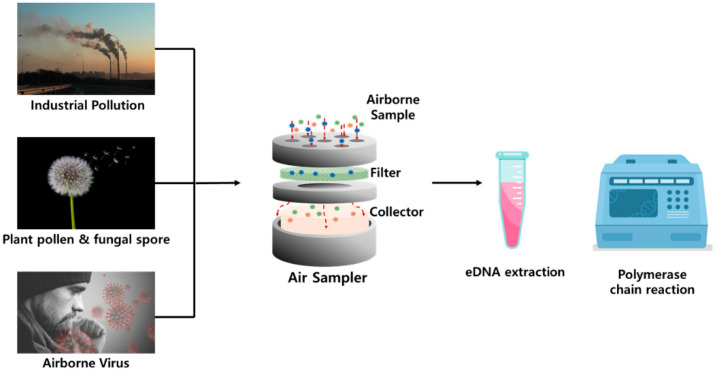
eDNA polymerase chain reaction progress in air.

Airborne eDNA monitoring is an emerging approach for detecting biological materials suspended in the atmosphere, offering a non-invasive means to assess microbial communities, pathogens, and macro-organismal biodiversity. The choice of sampling method significantly impacts the efficiency and reliability of downstream molecular analyses, with various techniques developed to suit different biological targets and environmental conditions (Figure 2, Table 1). Filter-based sampling is the most used approach due to its simplicity and high particle retention. Air is passed through membranes such as high-efficiency particulate air (HEPA) or polytetrafluoroethylene (PTFE) filters [46,47], which efficiently trap airborne particles, including microbial cells and extracellular DNA. This method is compatible with standard DNA extraction and sequencing protocols, making it ideal for microbial profiling and pathogen detection. However, prolonged use may lead to filter clogging and DNA degradation, necessitating careful control of airflow and storage conditions. Liquid impingers, which collect particles in a liquid medium, help preserve fragile or low-concentration targets such as viruses [48]. These are compatible with culture-based assays and are widely used in public health surveillance, though their field utility is limited by evaporation, equipment complexity, and maintenance needs. Cyclone samplers, using centrifugal force, collect particles onto solid or liquid substrates [49], providing high collection efficiency across a wide particle size range. While effective for larger targets such as pollen or insect DNA, they may cause mechanical stress that affects DNA quality and complicates extraction. Electrostatic precipitation systems capture charged particles on conductive surfaces using electrostatic attraction [50,51,52], thereby minimizing physical damage and preserving DNA integrity. Although suitable for real-time monitoring, their performance can be influenced by environmental factors such as humidity and particle charge. Passive sampling, which relies on natural particle deposition [53], is cost-effective and requires no power, making it useful for long-term or large-scale surveillance. However, its lower efficiency and vulnerability to environmental variability limit its applicability in high-sensitivity or quantitative studies.

Each method should be selected based on study goals. For example, filter-based and liquid impinger systems are optimal for detecting microbial and viral DNA, whereas cy-clone samplers are more effective for capturing macro-organismal DNA. Electrostatic methods offer advantages for ultrafine particle detection, while passive sampling, despite its limitations, provides valuable background ecological information. Nonetheless, challenges remain in airborne eDNA research, including low DNA yields due to sparse airborne biomass, degradation from environmental exposure, and spatiotemporal variability affected by weather conditions. Contamination control and high-sensitivity amplification protocols are essential for reliable analysis. Moreover, interpreting airborne eDNA signals is complex, as they may not directly reflect actual species presence or abundance. Integrating eDNA data with traditional biological surveys and atmospheric modeling is crucial to improving interpretation accuracy. Additionally, the atmospheric transport and vertical distribution of airborne eDNA particles are increasingly recognized as important factors influencing sampling results. Modeling the dispersion of eDNA using atmospheric transport models can help predict the spatial fate of biological particles under varying wind, humidity, and temperature conditions. These models are particularly valuable in urban or industrial areas where airflow is highly heterogeneous. Furthermore, stratification of airborne eDNA by vertical sampling height has been observed in several studies. For instance, microbial and plant eDNA concentrations may vary significantly between ground level, rooftop level, and higher atmospheric layers due to gravitational settling, particle size, and local airflow dynamics. Sampling at multiple vertical levels can therefore improve ecological resolution and help distinguish local vs. regional sources of eDNA. Technological advancements such as portable sampling devices, automated filtration systems, real-time sequencing technologies, and machine learning–based data analysis are rapidly expanding the capabilities of airborne eDNA monitoring. As protocols become increasingly standardized, this approach holds strong potential for applications in environmental surveillance, biodiversity conservation, biosecurity, and public health [54].

**Table 1:** eDNA sampling method in airborne target.

Category	Target	Sampling Methods	Ref.
Pollution	Microbial contamination (pathogens, fungi, viruses)	-Filter-based sampling (HEPA, PTFE filters, etc.) -Liquid impinger -Cyclone sampler	[46,47,48]
	Microbial community shifts due to industrial emissions	-Filter-based sampling -Passive sampling (settling method)	[49,53]
	Antibiotic-resistant bacteria monitoring	-Filter-based sampling -Airborne microbial trap	[50,51]
	Microbial responses to heavy metals/chemical pollutants	-Filter-based sampling -Passive sampling -Electrostatic precipitation	[49,50,53]
Ecosystem	Airborne insect eDNA (endangered and invasive species)	-Cyclone sampler -Filter-based sampling -Passive sampling	[49,53]
	Plant pollen and fungal spore distribution	-Filter-based sampling -Cyclone sampler -Passive sampling (settled dust collection)	[49,53]
	Seasonal ecological changes	-Passive sampling (long-term monitoring) -Filter-based sampling	[49,51,52,53]
Public Health and Biosecurity	Airborne virus surveillance (SARS-CoV-2, influenza, etc.)	-Liquid impinger -Electrostatic precipitation -Filter-based sampling	[49,50,53]
	Bioterrorism agent detection (anthrax, viral pathogens, etc.)	-Cyclone sampler -Electrostatic precipitation -Filter-based sampling	[49]
	Pathogen transmission monitoring in hospitals and public spaces	-Filter-based sampling -Liquid impinger -Airborne particle collector	[49,53]
Agriculture and Food Safety	Crop pathogen and pest surveillance	-Cyclone sampler -Passive sampling	[49,53]
	Monitoring pesticide-resistant airborne microbes	-Filter-based sampling -Electrostatic precipitation	[51,52]
	Early detection of livestock infectious diseases	-Liquid impinger -Filter-based sampling	[49,53]

#### 2.1.3. Applications and Case Studies

The application of airborne eDNA has garnered increasing attention as a non-invasive and high-resolution approach for monitoring biological signatures in the atmosphere [55]. Recent advances in sampling methodologies and analytical techniques have enabled their use in diverse contexts, ranging from biodiversity assessments to air quality monitoring and pathogen surveillance. These case studies underscore the versatility of airborne eDNA as an emerging tool in environmental and public health research.

One of the most promising applications of airborne eDNA is in atmospheric bioindicator-based pollution monitoring. Studies have demonstrated that microbial communities present in air samples undergo compositional shifts in response to environmental pollutants, including particulate matter (PM2.5 and PM10), nitrogen oxides, and heavy metals [33,34,35]. By characterizing the taxonomic and functional profiles of these airborne microbial assemblages, researchers have proposed eDNA-based bioindicators for assessing air quality and anthropogenic pollution impacts [38,49]. This approach offers a complementary method to conventional physicochemical air quality measurements, potentially providing a more comprehensive understanding of pollutant-induced biological responses in the atmosphere.

Beyond pollution assessments, airborne eDNA has shown significant utility in biodiversity monitoring and ecological research. DNA fragments from a wide range of taxa, including plants, insects, birds, and even mammals, have been successfully detected in air samples, demonstrating the feasibility of eDNA for passive species surveillance [56]. This non-invasive methodology allows for broad-scale monitoring of biodiversity patterns, species distributions, and migration events, reducing the need for direct observation or invasive sampling. Recent studies have highlighted its potential for conservation efforts, particularly in remote or inaccessible environments where traditional survey techniques are impractical [57].

Airborne eDNA has also emerged as a valuable tool in pathogen detection and epidemiological studies, particularly in the context of airborne disease transmission [50]. During the COVID-19 pandemic, researchers explored the detection of SARS-CoV-2 RNA in air samples from hospitals and public spaces, providing insights into viral dispersal patterns and potential transmission hotspots [50,51]. Similarly, airborne fungal spores linked to respiratory illnesses, such as those from *Aspergillus* and *Histoplasma* species, have been monitored using eDNA-based approaches, contributing to early warning systems for public health interventions [58]. The ability to track airborne pathogens in real-time underscores the potential of eDNA for strengthening disease surveillance and outbreak response strategies.

Additionally, airborne eDNA has been increasingly applied in climate change and atmospheric microbial ecology studies [52]. Long-term monitoring of airborne microbial communities has provided insights into the influence of climatic factors such as temperature, humidity, and wind patterns on microbial dispersal and ecosystem connectivity. Such studies contribute to a broader understanding of how airborne microbial dynamics respond to global environmental changes, offering critical data for climate impact assessments.

Collectively, these applications highlight the growing relevance of airborne eDNA in environmental research. As advancements in sampling efficiency, DNA extraction protocols, and high-throughput sequencing technologies continue to refine detection capabilities, airborne eDNA is poised to become an integral tool in environmental monitoring, biodiversity conservation, and public health surveillance [59,60]. Future research should focus on standardizing methodologies, optimizing data interpretation frameworks, and expanding the scope of airborne eDNA studies to enhance its applicability across disciplines.

#### 2.1.4. Challenges and Outlook

Airborne environmental DNA (eDNA) monitoring presents a promising approach for biodiversity assessment and ecological studies. However, several challenges must be addressed to realize its full potential. One major obstacle is the lack of standardized sampling protocols. Unlike aquatic or soil eDNA sampling, airborne eDNA collection is highly variable, with differences in filter materials, airflow rates, and collection devices affecting data comparability. Additionally, the degradation rates of airborne eDNA remain poorly understood, complicating efforts to determine optimal sampling conditions and timeframes.

A further challenge lies in distinguishing target eDNA from background noise. Airborne eDNA samples contain DNA from diverse sources, including microbial, fungal, plant, and animal material. Differentiating target species from background DNA requires advanced bioinformatics tools to ensure specificity and reduce false-positive detections. At the same time, low DNA concentrations, environmental degradation, or technical limitations can lead to false-negative detections, where target organisms are present but remain undetected. Moreover, data interpretation remains complex, as airborne eDNA datasets encompass DNA fragments from various taxa at different levels of abundance and degradation. Determining ecological relevance and distinguishing transient signals from resident species pose significant analytical difficulties.

Compounding these issues is the differential degradation rate of airborne eDNA depending on its biological origin. DNA from bacterial or fungal spores may be more resistant to environmental stressors (UV radiation, desiccation, oxidation) compared to that from plant pollen or animal cells, which tends to degrade faster. This taxon-specific degradation affects detection probability and can bias biodiversity assessments if not properly accounted for. Further research is needed to characterize the stability of airborne DNA from different taxonomic groups under varying environmental conditions to improve sampling design and interpretation.

Despite these challenges, advancements in technology and methodology offer promising solutions. The development of standardized protocols for airborne eDNA collection, filtration, and processing will enhance comparability across studies and enable broader applications in environmental monitoring. Additionally, further research into the persistence and degradation mechanisms of airborne eDNA under varying environmental conditions will inform optimal sampling strategies and improve detection accuracy. Integrating NGS, digital PCR (dPCR), and machine learning-based bioinformatics pipelines will refine detection methods and increase sensitivity and specificity while minimizing false positives.

Moreover, establishing global eDNA databases will facilitate data sharing and comparative analyses across regions and ecosystems. Such repositories will strengthen the role of eDNA as a tool for biodiversity monitoring, conservation biology, and epidemiological surveillance. As technological advancements continue, airborne eDNA monitoring is expected to evolve into a robust, non-invasive, and scalable method for detecting biodiversity in a rapidly changing world.

### 2.2. eDNA Monitoring in Soil, Sediment, and Groundwater

#### 2.2.1. The Importance of Terrestrial and Subsurface Environments

Terrestrial (soil, sediment) and subsurface (groundwater) environments play a critical role in maintaining the stability and function of ecosystems. Soils and sediments are responsible for cycling key nutrients, including nitrogen, carbon, and phosphorus, which are vital for maintaining ecosystem productivity and balance. Additionally, soil and groundwater function as natural filters for water storage and purification, contributing to flood control and water quality protection.

Pollutants, such as heavy metals, pesticides, and fertilizers, introduced through agricultural and industrial activities, accumulate in soil and sediments or seep into groundwater, potentially posing negative impacts on ecosystems and human health [61,62,63,64]. However, these environments also harbor microbial communities that naturally degrade or dilute pollutants, playing a crucial role in environmental remediation. For example, specific microorganisms can break down organic compounds or petroleum, aiding in the restoration of soil and groundwater [65].

In conclusion, surface and subsurface environments are central to the flow of materials and energy within the biosphere and are essential components in sustaining the sustainability of human and natural ecosystems [66,67]. Monitoring these environments is vital for pollution detection and the design of remediation strategies. eDNA-based technologies have emerged as innovative tools capable of supporting these efforts effectively. Several studies have identified eDNA metabarcoding as a promising tool for bridging knowledge gaps in soil biodiversity [56]. eDNA extracted from bulk samples such as soil and feces offers the potential to capture the DNA of multiple organisms and species simultaneously [68]. In fact, soil eDNA samples are gaining attention as a potentially valuable tool for assessing plant diversity, as they can contain signals from both above- and below-ground sources (e.g., pollen, debris, and roots) and DNA from both active and dormant plant tissues [69].

#### 2.2.2. Soil, Sediment, and Groundwater eDNA Sampling

Soil sampling is an essential process for environmental monitoring and pollution assessment, utilizing various methods to reflect the spatial heterogeneity of soils and obtain accurate data. One common method is core sampling, where soil core or drilling equipment is used to collect cylindrical samples at specific depths, allowing for the analysis of vertical soil layers and deep contamination levels [70]. This method is suitable for assessing the distribution of heavy metals at industrial sites or the penetration depth of fertilizers in agricultural lands. Additionally, the use of sampling tubes can minimize the loss of volatile substances during transport [71]. Composite sampling, which involves mixing small samples from multiple points to create a representative sample, is useful for evaluating the average condition of large areas or conducting preliminary surveys [72].

Surface sampling is appropriate for detecting fertilizer residues or initial contamination levels, while depth-specific sampling allows for the analysis of pollutant movement or vertical distribution by collecting individual samples from various depths. Dry and wet sampling methods are selected based on soil conditions, with dry soils suitable for chemical analysis and wet soils more appropriate for studying microbial communities or DNA preservation [73,74].

These diverse soil sampling methods and procedures enhance the quality of eDNA analysis, providing reliable data to accurately assess contamination levels and ecological changes.

Sediment sampling plays a crucial role in evaluating environmental pollution and ecological changes in aquatic environments by analyzing particles and materials deposited in water bodies. Sediment samples are typically collected from rivers, lakes, oceans, and wetlands and categorized into surface and deep sediments. Grab sampling is commonly used to assess the current state of surface pollutants or recent deposition trends. The relatively low penetration depth (3–30 cm) and high sample volume (0.5–75 L) of grab sampling make it particularly well-suited for the collection of surface sediments [75]. Deep sediment sampling involves using core sampling equipment to collect continuous layers of sediment, enabling the analysis of past contamination records and long-term deposition trends [76]. This approach is particularly useful for tracking temporal changes in pollutants such as heavy metals, organic matter, or microplastics deposited by industrial activities or agriculture. Moreover, DNA is well-preserved in anoxic conditions where oxygen levels are low, making deep sediment particularly valuable for eDNA studies [77]. Sediment sampling also requires consideration of the physical characteristics of the sampling site, such as water velocity, depth, and temperature, as well as sediment particle size and organic matter content, which can influence the analysis results. Sediment sampling provides vital information for understanding the complex pollution patterns and biological diversity of aquatic ecosystems, playing an important role in pollution remediation planning and ecological monitoring [78].

Groundwater sampling is a critical process for environmental monitoring, contamination assessment, and ecological studies, providing insights into subsurface conditions. Among the common methods, borehole or well sampling involves pumping groundwater to the surface for analysis [79,80]. To ensure that the extracted water accurately represents the aquifer, purging is conducted prior to sampling [81]. Typically, three to five well volumes of water are removed, or stabilization parameters such as pH, dissolved oxygen, and turbidity are monitored until they stabilize [82,83]. Controlling the flow rate during pumping is essential to avoid disturbing the aquifer’s structure, which could alter the sample’s properties. Additionally, strict measures must be taken to prevent cross-contamination between sampling sites, including the use of sterilized or disposable equipment and adherence to meticulous handling protocols.

Filtration is a fundamental step in all eDNA sampling, especially for groundwater eDNA analysis, as it concentrates DNA from the water. Filtration typically uses micro-sized pores to capture free-floating DNA, microbial cells, and DNA bound to particles [84,85,86]. Filtration is usually performed in the field using portable equipment to minimize DNA degradation, and filters are preserved immediately after collection by freezing or using chemical preservatives to maintain DNA integrity until laboratory analysis [84,87]. Proper storage and transport of groundwater samples are critical, with samples often maintained at low temperatures to preserve their chemical and biological composition.

**Figure 3:**
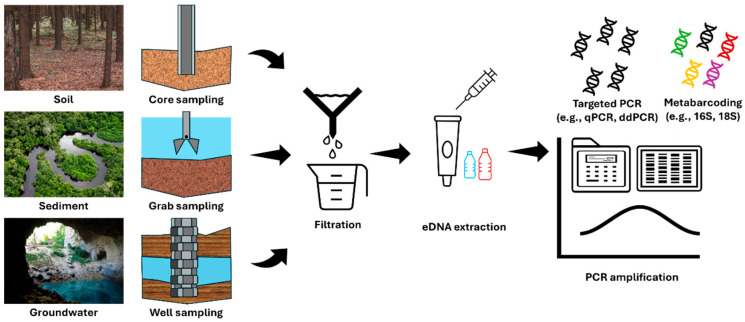
Soil, sediment, and groundwater eDNA polymerase chain reaction process.

After sampling, eDNA is amplified and processed through appropriate analytical procedures depending on the study’s objectives (Figure 3). During this process, it is essential to store samples at low temperatures to prevent DNA degradation and chemical changes and to collect only the necessary volume based on the sediment’s characteristics. Additionally, sampling tools must be thoroughly disinfected to prevent cross-contamination between samples. The advantages and disadvantages of each sampling method are summarized in Table 2.

#### 2.2.3. Pollution Monitoring and Biodegradation Studies

The use of eDNA in pollution monitoring and biodegradation studies has rapidly advanced, providing a non-invasive and highly sensitive method for tracking environmental health and microbial activity. eDNA allows for the detection of specific microbial species that can metabolize pollutants, offering insights into the pollution levels and the presence of microorganisms capable of degrading harmful substances. For instance, certain bacteria possess specialized enzymes that enable them to break down complex pollutants such as polychlorinated biphenyls (PCBs) or hydrocarbons [88,89]. By analyzing the eDNA of these microorganisms, researchers can assess the extent of pollution in each area without the need for traditional, more invasive sampling methods.

eDNA has been increasingly used in monitoring various types of pollution, from heavy metals to organic contaminants. The presence of pollutant-degrading microbes, which utilize pollutants such as petroleum hydrocarbons, pesticides, and plastics as carbon and energy sources [90,91], serves as a biomarker for pollution levels. By analyzing the diversity and activity of these microbial populations through eDNA, scientists can gain a deeper understanding of the processes that contribute to pollutant degradation [92,93]. The study of biodegradation processes through eDNA allows for the identification of key microbial species involved in pollutant breakdown, as well as the genes responsible for these metabolic activities.

An emerging area of concern in biodegradation studies is the spread of antibiotic resistance genes (ARGs), particularly in environments contaminated by agricultural runoff, wastewater, or industrial effluents. eDNA screening has proven effective in monitoring the presence and distribution of ARGs in various environmental matrices, including soil, groundwater, and sediment [93]. By tracking ARGs through eDNA, researchers can assess the risks of resistance spreading in ecosystems that are being treated for pollution. This understanding helps in designing strategies that address both pollutant degradation and the control of antibiotic resistance, ensuring that bioremediation efforts do not inadvertently contribute to the spread of resistance.

#### 2.2.4. eDNA Monitoring Case Studies in Soil, Sediment, and Groundwater

eDNA has emerged as a significant tool for monitoring and assessing the health of ecosystems impacted by anthropogenic activities. A prime example of this application is in the context of industrial brownfields, where eDNA analyses have been employed to evaluate microbial communities in sites contaminated by heavy metals and organic pollutants [94,95]. By identifying the presence and diversity of microorganisms, eDNA offers valuable insights into the biological processes occurring within these contaminated environments, thereby informing the development of effective remediation strategies aimed at mitigating pollution and restoring ecosystem function.

In the domain of agricultural soils, eDNA analysis has been utilized to examine the effects of fertilizer and pesticide applications on soil microbial diversity [96,97,98]. Studies have demonstrated that the use of these agricultural chemicals can lead to significant shifts in microbial populations, which may, in turn, influence soil fertility and crop productivity. Through monitoring microbial changes, eDNA provides crucial data that can guide the formulation of sustainable agricultural practices aimed at minimizing environmental degradation while maintaining agricultural productivity.

Furthermore, in the field of wetland restoration, eDNA has proven instrumental in tracking the recolonization of native flora and fauna following restoration efforts [99,100]. Sediment eDNA has been used to monitor the re-establishment of critical plant and animal species, as well as the recovery of beneficial microorganisms that contribute to ecosystem stability and nutrient cycling. The ongoing use of eDNA in these restoration projects enables continuous evaluation of the success of restoration efforts, facilitating adaptive management strategies that enhance biodiversity and ecosystem resilience.

These case studies underscore the versatility and efficacy of eDNA as a non-invasive, high-resolution method for assessing environmental health across various contexts. Its ability to provide comprehensive, real-time data makes it an invaluable tool for informing management and conservation strategies aimed at promoting sustainable environmental practices. Various case studies are summarized in Table 3.

**Table 3:** eDNA monitoring case studies of soil, sediment, and groundwater systems.

Case Study	Sampling Methods	Region	Analysis Method	Ref.
Industrial Brownfields	Surface	France	-PCR amplification (fungal 18S), NGS (Illumina MiSeq), OTU-based analysis	[92]
	Core	USA	-PCR amplification (16S, 18S, ITS), metabarcoding, ASV inference (DADA2), NGS (MiSeq)	[94]
	Surface	Australia	-PCR amplification (16S V4, 18S V7), metabarcoding, OTU clustering (Greenfield pipeline), NGS (MiSeq)	[95]
	Core	Italy	-PCR amplification (16S, 18S, COI), metabarcoding, ASV inference (QIIME2), NGS (MiSeq)	[100]
	Surface	Australia	-PCR amplification (16S, 18S), metabarcoding, OTU clustering (Greenfield), GS (MiSeq)	[101]
Agricultural Soil	Surface	China	-PCR-based metabarcoding (16S, 18S, ITS, COI), OTU clustering (97%), NGS (MiSeq)	[97]
	Core	Italy	-PCR-based metabarcoding (COI, 18S), ASV inference (DADA2 in QIIME2), NGS (MiSeq)	[98]
	Core	Germany	-PCR-based metabarcoding (COI for arthropods, D2 for fungi), OTU clustering (97%), NGS (MiSeq)	[102]
	Core	Denmark	-PCR-based metabarcoding (16S, ITS, 18S), OTU clustering (97%), NGS	[103]
	Surface	Germany	-eDNA metabarcoding for AM fungi, decomposers, and protists; ASV/OTU-based analysis; NGS (HiSeq, 454)	[104]
Wetland Restoration	Core	Australia	-PCR amplification (18S, trnL), OTU clustering (97%), NGS (Roche 454)	[105]
	Surface	USA	-PCR-based metabarcoding (vertebrate 12S), ASV inference, NGS (HiSeq)	[106]
	Surface	Canada	-Species-specific qPCR assays (northern leopard frog, boreal chorus frog)	[107]
	Surface	China	-PCR amplification (18S V4), NGS (Illumina MiSeq)	[108]

#### 2.2.5. Key Considerations

When utilizing eDNA for monitoring soil, sediment, and groundwater, several critical factors must be considered to ensure accurate interpretation of results. Two major aspects that influence eDNA analysis in these environments are DNA adsorption and temporal resolution.

Soil and sediment particles have a strong capacity to bind DNA, which can introduce complexities in extraction and downstream analysis. The adsorption of DNA onto mineral surfaces, organic matter, and clay particles can reduce DNA yield, potentially leading to underestimation of biodiversity or species presence [109,110]. Additionally, adsorbed DNA may persist for extended periods, acting as “legacy DNA” that reflects historical biological communities rather than current ones. This can complicate ecological assessments, particularly in environments with fluctuating conditions that may release previously bound DNA back into solution. To mitigate these challenges, optimized DNA extraction protocols and careful selection of chemical and enzymatic treatments are essential to improve recovery and reduce biases associated with adsorption.

The persistence of eDNA in soils and sediment poses challenges in distinguishing between contemporary and past biological communities. Unlike in aquatic environments, where DNA degrades more rapidly, terrestrial and sedimentary eDNA can remain detectable for prolonged periods, potentially leading to misinterpretations of species presence. This is because ultraviolet light has a relatively harder time penetrating the soil, which slows down the degradation of eDNA [111,112]. Previous studies have confirmed that eDNA can still be detected up to 12 weeks after being exposed to the environment [113]. Proper timing of sampling, in conjunction with degradation studies and quantitative assessments, can help differentiate between recent and historical signals. Furthermore, integrating eDNA data with other ecological and environmental parameters can enhance the reliability of temporal assessments.

Addressing these key considerations is essential for improving the accuracy and reliability of eDNA-based studies in soil, sediment, and groundwater environments. By accounting for DNA adsorption and temporal resolution, researchers can enhance their ability to track biodiversity, assess ecosystem changes, and support informed conservation and management decisions.

### 2.3. eDNA Monitoring in Water Systems

#### 2.3.1. Value of Aquatic eDNA

**Figure 4:**
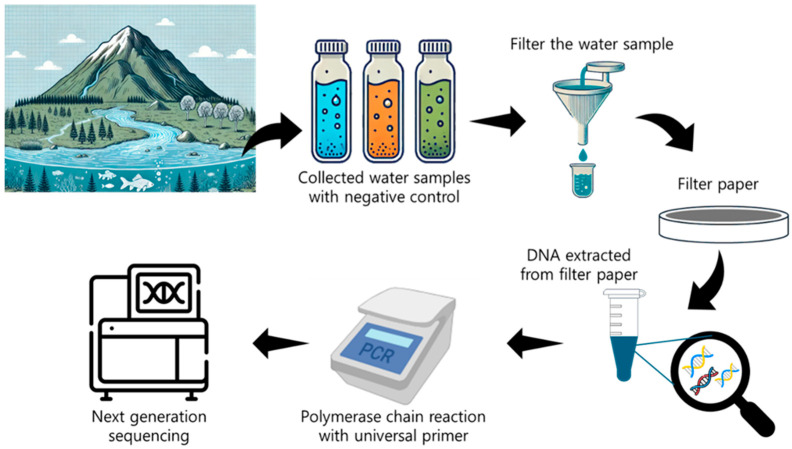
Workflow for detection of aquatic species using eDNA.

Aquatic systems provide an ideal medium for eDNA capture because water disperses and preserves genetic material shed by organisms (Figure 4) [114]. This enables the detection of resident and transient species across habitats from streams and lakes to coastal zones and oceans. Water’s fluidity and connectivity distribute genetic markers widely, allowing assessment of species richness, early detection of invasive or endangered species, and monitoring of ecosystem health [56,115]. Aquatic eDNA also detects low-density or elusive organisms beyond the reach of nets or visual surveys [114,116].

In addition, specific genetic markers reveal contaminants or pathogens for pollution monitoring [117,118]. These analyses offer snapshots of biodiversity and insights into ecological and temporal dynamics. By combining traditional surveys with molecular data, aquatic eDNA supports informed conservation, resource management, and policy decisions [119,120]. Ongoing advances in sequencing and bioinformatics will expand its role as an essential tool in environmental monitoring [121].

#### 2.3.2. eDNA Sampling Approaches

The effectiveness of aquatic eDNA studies is largely dependent on the sampling approaches employed, which have evolved to capture the intricacies of waterborne genetic material (Table 4) [109]. One common method is the use of grab samples, where water is collected in sterile containers at specific points in time [122,123]. This method is particularly useful for obtaining a snapshot of the eDNA present in a water body at a given moment, which can be crucial for studies requiring rapid assessment or those focused on short-term ecological events. However, while grab samples offer convenience and simplicity, they may not fully represent the temporal variability in eDNA distribution, especially in dynamic systems where environmental conditions fluctuate throughout the day or seasonally. To address this limitation, continuous or automated sampling techniques have been developed. These methods employ automated samplers that collect water at regular intervals over extended periods, providing a more comprehensive picture of the temporal dynamics of eDNA. Continuous sampling is particularly valuable in large-scale monitoring programs or in environments where changes occur rapidly and may be missed by sporadic sampling. In both cases, filtration and concentration are critical steps that follow water collection. Typically, water is passed through filters with pore sizes ranging from 0.2 µm to 0.45 µm to capture eDNA-laden particles [124]. The volume of water filtered can be adjusted based on the specific objectives of the study and the presumed abundance of target eDNA, with larger volumes often required in environments with low DNA concentrations. The captured material is then processed in the laboratory for DNA extraction, amplification, and sequencing. This multi-step approach not only increases the likelihood of detecting rare or low-abundance species but also minimizes potential contamination. Advances in filtration technology and automated sampling have significantly enhanced the reliability and reproducibility of aquatic eDNA studies. As a result, these sampling approaches have become integral to the robust application of eDNA monitoring in diverse aquatic settings, ensuring that the genetic data collected is both representative and actionable for subsequent ecological analyses.

**Table 4:** eDNA sampling methods of water systems.

Sampling Method	Advantages	Limitations	Typical Applications	Analysis Method	Ref.
Grab sampling	-Simple and cost-effective -Quick to perform -Minimal equipment required	-Represents only a single time point -May miss temporal variation	-Rapid assessments -Preliminary surveys -Small-scale studies	Two-step PCR, metabarcoding	[122,125]
Continuous/ Automated Sampling	-Captures temporal variability -Generates high-resolution datasets -Reduces human error during collection	-Higher cost and maintenance requirements -More complex data management and analysis	-Long-term monitoring programs -Dynamic environments -Detailed temporal studies		[126,127]
Passive Sampling	-Low power requirements -Can be deployed in hard-to-access or remote areas -Minimizes disturbance during sampling	-May have lower control over sampling timing -Potential variability in accumulation rates depending on environmental factors	-Environments with limited human access -Long-term or continuous eDNA accumulation studies		[61,125,128,129,130]
Remote and Autonomous Sampling	-Enables sampling in remote, harsh, or hazardous environments -Real-time data transmission and monitoring -Reduces the need for frequent human intervention	-Requires significant initial investment -Dependence on reliable power sources and communication infrastructure	-Remote locations (e.g., high-altitude streams, polar regions) -Real-time environmental monitoring applications	PCR, qPCR, NGS	[131]

#### 2.3.3. Wastewater Surveillance

Wastewater surveillance represents an innovative application of eDNA technology, offering a large-scale approach to monitor public health and environmental quality [55,132]. As a complex matrix of human and animal excreta and various contaminants, wastewater provides genetic material that reveals pathogens, antibiotic resistance genes, and other biological markers [133,134]. Wastewater-based epidemiology has monitored viruses such as SARS-CoV-2 and norovirus, enabling tracking of community infection dynamics in near real-time for early intervention [135,136]. High-throughput sequencing profiles the genetic diversity in wastewater samples, revealing the distribution and potential sources of resistance genes [137]. Profiling resistance genes is vital for understanding antimicrobial resistance spread and its risks to human health and ecosystems [138,139,140]. Real-time monitoring can inform targeted interventions, guide sanitation and waste treatment protocols, and support effective public health strategies. Wastewater surveillance can also extend to monitoring chemical pollutants and emerging contaminants. The continuous flow of genetic data serves as an early warning system for public health threats and provides insight into the ecological impacts of urbanization and industrial activity. As methodologies improve and standardized protocols are established, wastewater surveillance will play a growing role in safeguarding public health and advancing our understanding of microbial ecosystems in urban settings. However, wastewater surveillance using eDNA faces several analytical challenges. One critical limitation is differentiating between viable and nonviable pathogens, as eDNA analysis alone does not provide direct evidence of pathogen viability or infectivity. Additionally, genetic material derived from humans may interfere with environmental microbial signals, complicating pathogen identification and quantification. Strategies such as integrating viability PCR assays or RNA-based methods, along with rigorous bioinformatics filtering approaches, are crucial to overcoming these limitations and enhancing the accuracy of wastewater-based epidemiology.

#### 2.3.4. Resource Water Bodies

Resource water bodies such as lakes, rivers, and coastal zones are critical reservoirs of biodiversity and natural resources, and eDNA monitoring in these systems has proven invaluable [107]. These water bodies provide drinking water, recreation, and economic resources and serve as habitats for myriad species. eDNA monitoring offers an efficient approach to assess ecosystem health and diversity. By sampling water from lakes, rivers, and coastal zones, researchers detect endemic and endangered species vital for ecological balance and identify invasive species early for intervention and management.

In addition to species detection, eDNA tracks pollutant degradation by microbial consortia following oil spills or chemical discharges [141,142]. This application aids in the assessment of natural remediation effectiveness and informs the design of enhanced bioremediation strategies [143]. Moreover, eDNA metabarcoding reveals community shifts in microbial abundance or diversity that indicate ecological stress from nutrient overloading, chemical pollutants, or climatic changes [144]. Quantitative and qualitative assessments of these shifts help scientists understand disturbance impacts and develop adaptive management strategies to mitigate negative outcomes. Thus, eDNA monitoring in resource water bodies plays a dual role in biodiversity conservation and sustainable resource management. It provides a sensitive and comprehensive approach to track ecological changes and ensures these critical water bodies continue to provide essential ecosystem services.

#### 2.3.5. eDNA Monitoring Applications and Case Studies in Water Systems

Case studies in aquatic eDNA monitoring highlight its practical and transformative applications across diverse environments. In freshwater systems, invasive carp have threatened native fish and disrupted ecosystems, often causing severe ecological and economic consequences. Through regular eDNA surveys, researchers detected carp genetic signatures well before population expansion, enabling proactive management measures, including targeted removal and habitat modifications, to limit spread and mitigate impacts.

In coastal coral reef monitoring [144], eDNA sampling from reef waters generated comprehensive profiles of fish communities and detected coral pathogens that signal disease outbreaks. This high-resolution method guided conservation planning, improved reef fisheries management, and supported restoration strategies under pressures from climate change, pollution, and overfishing. eDNA monitoring also advances harmful algal bloom prediction. Blooms driven by nutrient loading and environmental shifts produce toxins that harm marine life, fisheries, and tourism. Tracking eDNA from toxin-producing algae allowed continuous monitoring of bloom dynamics and early prediction of outbreaks, which is vital for issuing public health warnings, deploying mitigation strategies, and reducing economic losses [145]. These cases demonstrate eDNA’s versatility and efficacy as a molecular monitoring tool. By delivering rapid, accurate, and cost-effective assessments of species presence, pathogen prevalence, and ecosystem health, eDNA applications in invasive species control, coral reef conservation, and algal bloom management are transforming environmental stewardship and public health protection in aquatic systems.

## 3. Future Directions

Environmental DNA (eDNA) research is at a pivotal point, offering transformative potential for revolutionizing environmental monitoring through technological innovation, methodological refinement, and interdisciplinary collaboration. As a non-invasive and highly sensitive tool for detecting species and assessing ecosystem health, eDNA has already provided valuable insights across air, water, soil, and sediment. However, fully harnessing its potential requires addressing persistent challenges and seizing new opportunities.

Although ISO guidelines (e.g., ISO TC 276 WG7) and minimum information for publication of quantitative real-time PCR experiments (MIQE) reporting standards provide detailed frameworks for sampling and DNA extraction, substantial variability remains across environmental matrices, preservation techniques, and laboratory workflows [146,147]. This variability necessitates continued, context-specific validation to ensure the reproducibility and comparability of eDNA datasets. Harmonizing protocols globally would enhance reproducibility and enable meta-analyses and large-scale ecological assessments essential for informing environmental policy and management.

Optimizing DNA recovery from complex matrices remains a priority. Inhibitors and particulate matter can bind to DNA and reduce detection sensitivity. Future research should focus on refining extraction protocols, chemical treatments, and mechanical processes to maximize yield, even from degraded or low-biomass samples. Furthermore, clearly defining downstream analytical approaches is essential to fully harness eDNA’s potential. Typical workflows include amplicon-based sequencing such as 16S and 18S rRNA gene metabarcoding, shotgun metagenomics for comprehensive taxonomic and functional profiling, and target-specific quantification using quantitative PCR (qPCR) or droplet digital PCR (ddPCR) [148,149,150]. Marker genes are selected based on taxa of interest, such as cytochrome oxidase I (COI) for metazoans, internal transcribed spacer (ITS) for fungi, and 12S for vertebrates [151,152,153]. Future methodological frameworks should standardize marker selection and data interpretation tailored to specific ecological and monitoring objectives. These improvements will enhance biodiversity assessments and pollutant tracking by improving data accuracy.

Technological innovation is driving eDNA toward real-time monitoring capabilities. Miniaturized sequencing devices and portable polymerase chain reaction (PCR) systems allow on-site analysis, reducing the time between sampling and interpretation. CRISPR-based detection systems are emerging as highly specific and sensitive tools for rapid target identification. Combined with automated samplers and remote sensing platforms, these technologies may soon support high-resolution environmental monitoring networks.

The integration of eDNA data with advanced bioinformatics pipelines and big data analytics is expected to significantly reshape ecological monitoring and biodiversity assessment [154]. As high-throughput sequencing becomes more accessible and cost-effective, eDNA metabarcoding datasets are rapidly growing and becoming complex [155]. These datasets often contain thousands of species-level operational taxonomic units or exact sequence variants, which pose analytical challenges for conventional taxonomic methods originally designed for small-scale datasets.

To meet these challenges, research is increasingly incorporating machine learning. Algorithms such as Random Forests, Support Vector Machines, and ensemble methods are well suited to model complex, nonlinear ecological relationships and can perform reliably even when data are noisy or incomplete [156].

As eDNA datasets scale up to include terabytes of sequencing data from multiple sites and time points, concerns about scalability and efficiency are growing. Machine learning frameworks offer solutions: supervised algorithms such as Random Forests are used for taxonomic assignments, while unsupervised models such as autoencoders identify anomalies in noisy datasets. One study showed that applying gradient boosting models directly to raw metabarcoding reads improved rare species detection by over 15% compared to traditional rule-based methods [23]. Similarly, Long Short-Term Memory (LSTM) neural networks have been used to forecast temporal shifts in coastal microbial eDNA communities, accurately predicting changes up to a month in advance [23].

Machine learning techniques also clarify how eDNA is transported, diluted, and degraded in natural systems, aiding the development of site-specific environmental indicators. Tools such as the Variable Selection Using Random Forests package help identify which environmental variables most influence eDNA detectability.

Beyond classification and regression, machine learning is used for dimensionality reduction, noise filtering, and pattern recognition [157]. Deep learning models such as convolutional neural networks and autoencoders can be trained on synthetic datasets to correct sequencing errors and estimate genetic diversity, even from ultra-short or degraded eDNA fragments [158]. These methods improve taxonomic resolution, particularly for underrepresented or poorly characterized taxa. Furthermore, the choice of bioinformatics pipelines significantly impacts species resolution and detection accuracy. Traditional Operational Taxonomic Unit (OTU)-based clustering methods group similar sequences based on a predefined similarity threshold, but this approach may obscure subtle genetic differences and reduce taxonomic resolution. Conversely, Amplicon Sequence Variant (ASV) methods, such as those implemented by the DADA2 pipeline, resolve unique sequences precisely, enabling improved detection and differentiation of closely related species or strains, even when analyzing ultra-short or degraded eDNA fragments. Future methodological improvements should carefully evaluate the trade-offs between OTU- and ASV-based analyses in different ecological contexts, ensuring optimal selection of bioinformatics tools tailored to study objectives.

In summary, eDNA is evolving from a basic detection tool into a comprehensive platform for predictive ecosystem monitoring. As environmental pressures intensify, the integration of machine learning will be essential for extracting actionable insights from increasingly complex datasets, enhancing conservation planning and ecosystem resilience.

Global standardization and data sharing are essential for advancing eDNA into routine monitoring. International consortia and centralized repositories can facilitate the development of best practices, promote open science, and enable large-scale comparative studies. For instance, the European Chemicals Agency already acknowledges standardized eDNA occurrence data in its environmental exposure guidance and has released protocols for incorporating molecular monitoring into chemical risk assessments [159].

Interdisciplinary collaboration across molecular biology, ecology, environmental engineering, and policy will be key to developing robust and applicable monitoring frameworks. Integrating eDNA with remote sensing, geographic information systems (GIS), and ecological modeling can generate high-resolution environmental change assessments and early warning systems for biodiversity loss, pollution events, and disease emergence. These comprehensive approaches support adaptive management by delivering timely and actionable insights.

Finally, future studies should investigate DNA degradation kinetics across variables such as temperature, pH, UV exposure, and microbial activity to improve interpretation accuracy. Research on the spatial heterogeneity of eDNA within ecosystems will also help refine sampling strategies to ensure local biodiversity is accurately represented.

Expanding eDNA applications into public health and biosecurity is another promising avenue. As urbanization and industrial activities increase the risk of pathogen spread and antibiotic resistance, eDNA can provide a non-invasive approach for early detection of microbial threats in both natural and built environments. Future research should focus on distinguishing viable pathogens from non-infectious genetic material to improve eDNA reliability in epidemiological surveillance and public health decision-making. As eDNA is increasingly applied in public health and biosecurity, ethical considerations must also be addressed. The unintentional capture of human genetic material raises concerns regarding privacy, consent, and data ownership, especially when samples are collected from public or urban environments. Clear guidelines and data governance frameworks will be essential to ensure responsible and transparent use of eDNA technologies [160].

## 4. Conclusions

Environmental DNA (eDNA) has revolutionized environmental monitoring by enabling non-invasive, high-resolution detection of biodiversity and ecological changes across diverse environments. Its integration with advanced sequencing and bioinformatics tools has significantly expanded its sensitivity and applicability. An eDNA analysis has revealed ecological patterns that were previously inaccessible through conventional methods, such as the detection of elusive amphibians in alpine streams, spatiotemporal tracking of airborne viral RNA in clinical settings, and diurnal shifts in plant and insect signals in forest ecosystems. Despite remaining challenges related to standardization, degradation, and data interpretation, continued technological progress is enhancing the accuracy, scalability, and utility of eDNA. As a result, it is increasingly recognized as a transformative tool for early warning, conservation planning, and ecosystem management, with growing relevance in both environmental and public health contexts. To support effective implementation, Table 5 outlines key stakeholder-specific recommendations for researchers, regulators, and conservation practitioners, promoting coordinated actions that align with the expanding role of eDNA in environmental and public health monitoring.

## Figures and Tables

**Table 2:** Soil, sediment, and groundwater sampling method.

Category	Methods	Advantages	Disadvantages
Soil	Core sampling	-Maintains stratigraphic continuity, allowing analysis of vertical pollutant distribution -Suitable for studying long-term environmental changes -Can collect deep layers, making it useful for geological and sedimentological research	-Requires complex and expensive equipment -Time-consuming sample collection process -Possible structural deformation in soft layers
	Grab sampling	-Fast and easy to perform -Cost-effective with simple equipment -Enables rapid sampling from multiple locations	-Limited to surface-level information -Cannot analyze subsurface contamination or historical changes -May does not represent deeper soil conditions accurately
Sediment	Core	-Provides information on vertical variations in soil composition and contaminants -Allows historical analysis of soil conditions -Essential for studying subsurface pollution migration	-Requires specialized equipment and can be time-consuming -More expensive than surface sampling -May disturb deeper soil layers during extraction
	Surface	-Quick and easy to perform -Requires minimal equipment and lower cost -Suitable for large-scale surface contamination assessment	-Limited to surface-level information -Cannot analyze subsurface contamination or historical changes -May does not represent deeper soil conditions accurately
Groundwater	Filtration	-Enhances eDNA analysis by concentrating DNA and microorganisms -Remove suspended solids, improving water quality for chemical analysis -Immediate field filtration minimizes sample degradation -Allows selection of various filter materials and pore sizes for optimized results	-Clog easily, especially with high particulate loads -DNA recovery efficiency may vary depending on filter type -Requires field equipment and careful contamination control -Time-consuming when processing large water volumes

**Table 5:** Key recommendations for stakeholders in eDNA monitoring.

Stakeholder	Key Recommendations
Researchers	- Prioritize the development and validation of standardized eDNA sampling, filtration, and preservation protocols to ensure data comparability across studies and regions. - Invest in the refinement of bioinformatics pipelines to enhance taxonomic resolution, reduce false positives, and improve detection sensitivity in complex environmental samples. - Promote interdisciplinary collaboration to integrate eDNA results with ecological modeling, population genetics, and remote sensing data.
Regulators and Policymakers	- Establish evidence-based regulatory frameworks for the incorporation of eDNA technologies into national and regional biodiversity monitoring programs. - Facilitate inter-agency coordination and data-sharing platforms to promote the effective use of eDNA in environmental assessment and reporting. - Allocate long-term funding to support routine eDNA monitoring as a cost-effective tool for regulatory compliance and early warning systems.
Conservationists	- Integrate eDNA surveillance into species conservation plans, particularly for rare, cryptic, or invasive taxa that are difficult to monitor using traditional methods. - Employ eDNA data to inform adaptive management strategies, such as habitat restoration, translocation, or eradication efforts. - Build capacity for in-field eDNA sampling and analysis through training programs and partnerships with research institutions.

## Abbreviation

AI	Artificial Intelligence
ARGs	Antibiotic Resistance Genes
ASV	Amplicon Sequence Variant
COI	Cytochrome Oxidase I
CRISPR-Cas	Clustered Regularly Interspaced Short Palindromic Repeats–CRISPR-associated proteins
dPCR	Digital Polymerase Chain Reaction
ddPCR	Droplet Digital Polymerase Chain Reaction
eDNA	Environmental DNA
GIS	Geographic Information Systems
ITS	Internal Transcribed Spacer
HEPA	High-Efficiency Particulate Air (filter)
HTS	High-Throughput Sequencing
ISO	International Organization for Standardization
LAMP	Loop-Mediated Isothermal Amplification
RPA	Recombinase Polymerase Amplification
LSTM	Long Short-Term Memory
MIQE	Minimum Information for Publication of Quantitative Real-Time PCR Experiments
NGS	Next Generation Sequencing
OTU	Operational Taxonomic Unit
PCBs	Polychlorinated Biphenyls
PCR	Polymerase Chain Reaction
PM	Particulate Matter
PTFE	Polytetrafluoroethylene
qPCR	Quantitative Polymerase Chain Reaction
US EPA	United States Environmental Protection Agency

## References

[1] Li J., Pei Y., Zhao S., Xiao R., Sang X., Zhang C. (2020). A review of remote sensing for environmental monitoring in China. Remote Sens..

[2] Estoque R.C., Murayama Y. (2017). Monitoring surface urban heat island formation in a tropical mountain city using Landsat data (1987–2015). ISPRS-J. Photogramm..

[3] Ullo S.L., Sinha G.R. (2020). Advances in smart environment monitoring systems using IoT and sensors. Sensors.

[4] Lindenmayer D.B., Likens G.E. (2010). The science and application of ecological monitoring. Biol. Conserv..

[5] Amann R.I., Ludwig W., Schleifer K.H. (1995). Phylogenetic identification and in situ detection of individual microbial cells without cultivation. Microbiol. Rev..

[6] Cole D.N. (1995). Experimental trampling of vegetation. I. Relationship between trampling intensity and vegetation response. J. Appl. Ecol..

[7] Rodriguez-Gil J.L., Sauto J.S.S., Gonzalez-Alonso S., Sanchez P.S., Valcarcel Y., Catala M. (2013). Development of cost-effective strategies for environmental monitoring of irrigated areas in mediterranean regions: Traditional and new approaches in a changing world. Agric. Ecosyst. Environ..

[8] Taberlet P., Coissac E., Hajibabaei M., Rieseberg L.H. (2012). Environmental DNA. Mol. Ecol..

[9] Thomsen P.F., Willerslev E. (2015). Environmental DNA—An emerging tool in conservation for monitoring past and present biodiversity. Biol. Conserv..

[10] Ficetola G.F., Taberlet P., Coissac E. (2016). How to limit false positives in environmental DNA and metabarcoding?. Mol. Ecol. Resour..

[11] Bohara K., Yadev A.K., Joshi P. (2022). Detection of fish pathogens in freshwater aquaculture using eDNA methods. Diversity.

[12] Klymus K.E., Richter C.A., Chapman D.C., Paukert C. (2015). Quantification of eDNA shedding rates from invasive bighead carp Hypophtalmichthys nobilis and silver carp Hypophthalmichthys molitrix. Biol. Conserv..

[13] Deiner K., Fronhofer E.A., Machler E., Walser J.C., Altermatt F. (2016). Environmental DNA reveals that rivers are conveyer belts of biodiversity information. Nat. Commun..

[14] Sahu A., Kumar N., Singh C.P., Singh M. (2023). Environmental DNA (eDNA): Powerful technique for biodiversity conservation. J. Nat. Conserv..

[15] Alfano N., Dayaram A., Axtner J., Tsangaras K., Kampmann M.L., Mohamed A., Wong S.T., Gilbert M.T., Wilting A., Greenwood A.D. (2021). Non-invasive surveys of mammalian viruses using environmental DNA. Methods Ecol. Evol..

[16] Callahan B.J., McMurdie P.J., Rosen M.J., Han A.W., Johnson A.J.A., Holmes S.P. (2016). DADA2: High-resolution sample inference from Illumina amplicon data. Nat. Methods.

[17] Melendy S.A., Olson J.R. (2024). Identifying Key Environmental Drivers of Reach-Scale Salmonid eDNA Recovery With Random Forest. Environ. DNA.

[18] Bauer D.C., Wilson L.O., Twine N.A. (2022). Artificial Intelligence in Medicine: Applications, Limitations and Future Directions. Artificial Intelligence in Medicine: Applications, Limitations and Future Directions.

[19] Hinz S., Coston-Guarini J., Marnane M., Guarini J.M. (2022). Evaluating eDNA for use within marine environmental impact assessment. J. Mark. Sci. Eng..

[20] Xiong F., Shu L., Zeng H., Gan X., He S., Peng Z. (2022). Methodology for fish biodiversity monitoring with environmental DNA metabarcoding: The primers, databases and bioinformatic pipelines. Water Biol. Secur..

[21] Mace B., Hocde R., Marques V., Guerin P.E., Valentini A., Arnal V., Pellissier L., Manel S. (2022). Evaluating bioinformatics pipelines for population-level inference using environmental DNA. Environ. DNA.

[22] Ding Y., Zhang F., Zhang J. (2023). Applicability and advantage of mitochondrial metagenomics and metabarcoding in spider biodiversity survey. Diversity.

[23] O’Donncha F., Hu Y., Palmes P., Burke M., Filgueira R., Grant J. (2022). A spatio-temporal LSTM model to forecast across multiple temporal and spatial scales. Ecol. Inform..

[24] Park K.S., Choi A., Kim H.J., Park I., Eom M.-S., Yeo S.-G., Son R.G., Park T.-I., Lee G., Soh H.T. (2023). Ultra-sensitive label-free SERS biosensor with high-throughput screened DNA aptamer for universal detection of SARS-CoV-2 variants from clinical samples. Biosens. Bioelectron..

[25] Toshiaki S.J. (2023). Utilizing the state of environmental DNA (eDNA) to incorporate time-scale information into eDNA analysis. Proc. R. Soc. B-Biol. Sci..

[26] Hempel C.A., Buchner D., Mack L., Brasseur M.V., Tulpan D., Leese F., Steinke D. (2023). Predicting environmental stressor levels with machine learning: A comparison between amplicon sequencing, metagenomics, and total RNA sequencing based on taxonomically assigned data. Front. Microbiol..

[27] Wang C., Chen L., Li X., Gu J., Xiang Y., Fang L., Chen L., Li Y. (2024). Development of an all-in-one real-time PCR assay for simultaneous detection of spotted fever group rickettsiae, severe fever with thrombocytopenia syndrome virus and hantaan virus prevalent in central China. PLoS Negl. Trop. Dis..

[28] Lu A., Liu H., Shi R., Cai Y., Ma J., Shao L., Rong V., Gkitsas N., Lei H., Highfill S.L. (2020). Application of droplet digital PCR for the detection of vector copy number in clinical CAR/TCR T cell products. J. Transl. Med..

[29] Zhang Y., Qiao S., Zeng Y., Gao D., Han N., Zhou J. (2021). CAE-CNN: Predicting transcription factor binding site with convolutional autoencoder and convolutional neural network. Expert Syst. Appl..

[30] Yan Z., Luo Y., Chen X., Yang L., Yao M. (2024). Angling and trolling for eDNA: A novel and effective approach for passive eDNA capture in natural waters. Environ. Int..

[31] Doi H., Watanabe T., Nishizawa N., Saito T., Nagata H., Kameda Y., Maki N., Ikeda K., Fukuzawa T. (2021). On-site environmental DNA detection of species using ultrarapid mobile PCR. Mol. Ecol. Resour..

[32] Zhao J., Xu H., Xu C., Yin W., Luo L., Liu G., Wang Y. (2025). Smartphone-integrated RPA-CRISPR-Cas12a Detection System with Microneedle Sampling for Point-of-Care Diagnosis of Potato Late Blight in Early Stage. bioRxiv.

[33] Su X., Sutarlie L., Loh X.J. (2020). Sensors and Analytical Technologies for Air Quality: Particulate Matters and Bioaerosols. Chem. Asian J..

[34] Yang C.T., Chen H.W., Chang E.J., Kristiani E., Nguyen K.L.P., Chang J.S. (2021). Current advances and future challenges of AIoT applications in particulate matters (PM) monitoring and control. J. Hazard. Mater..

[35] Liu X., Jayaratne R., Thai P., Kuhn T., Zing I., Christensen B., Lamont R., Dunbabin M., Zhu S., Gao J. (2020). Low-cost sensors as an alternative for long-term air quality monitoring. Environ. Res..

[36] Petruci J.F.d.S., Barreto D.N., Dias M.A., Felix E.P., Cardoso A.A. (2022). Analytical methods applied for ozone gas detection: A review. Trends Anal. Chem..

[37] Yeo M.J., Kim Y.P. (2021). Long-term trends of surface ozone in Korea. J. Clean. Prod..

[38] Thangamani G.J., Pasha S.K.K. (2021). Titanium dioxide (TiO(2)) nanoparticles reinforced polyvinyl formal (PVF) nanocomposites as chemiresistive gas sensor for sulfur dioxide (SO(2)) monitoring. Chemosphere.

[39] Idrees Z., Zheng L. (2020). Low cost air pollution monitoring systems: A review of protocols and enabling technologies. J. Ind. Inf. Integr..

[40] Vîrghileanu M., Săvulescu I., Mihai B.-A., Nistor C., Dobre R. (2020). Nitrogen Dioxide (NO2) Pollution Monitoring with Sentinel-5P Satellite Imagery over Europe during the Coronavirus Pandemic Outbreak. Remote Sens..

[41] Huang J., Wang D., Zhu Y., Yang Z., Yao M., Shi X., An T., Zhang Q., Huang C., Bi X. (2024). An overview for monitoring and prediction of pathogenic microorganisms in the atmosphere. Fundam. Res..

[42] Mahaffee W.F., Margairaz F., Ulmer L., Bailey B.N., Stoll R. (2023). Catching Spores: Linking Epidemiology, Pathogen Biology, and Physics to Ground-Based Airborne Inoculum Monitoring. Plant Dis..

[43] Suanno C., Aloisi I., Fernandez-Gonzalez D., Del Duca S. (2021). Monitoring techniques for pollen allergy risk assessment. Environ. Res..

[44] Khatib M., Haick H. (2022). Sensors for Volatile Organic Compounds. ACS Nano.

[45] Lynggaard C., Bertelsen M.F., Jensen C.V., Johnson M.S., Froslev T.G., Olsen M.T., Bohmann K. (2022). Airborne environmental DNA for terrestrial vertebrate community monitoring. Curr. Biol..

[46] Roger F., Ghanavi H.R., Danielsson N., Wahlberg N., Löndahl J., Pettersson L.B., Andersson G.K.S., Boke Olén N., Clough Y. (2022). Airborne environmental DNA metabarcoding for the monitoring of terrestrial insects—A proof of concept from the field. Environ. DNA.

[47] Sullivan A.R., Karlsson E., Svensson D., Brindefalk B., Villegas J.A., Mikko A., Bellieny D., Siddique A.B., Johansson A.-M., Grahn H. (2023). Airborne eDNA captures three decades of ecosystem biodiversity. bioRxiv.

[48] Goray M., Taylor D., Bibbo E., Fantinato C., Fonnelop A.E., Gill P., van Oorschot R.A.H. (2024). Emerging use of air eDNA and its application to forensic investigations—A review. Electrophoresis.

[49] Polling M., Buij R., Laros I., de Groot G.A. (2024). Continuous daily sampling of airborne eDNA detects all vertebrate species identified by camera traps. Environ. DNA.

[50] Yan S., Liu Q., Liang B., Zhang M., Chen W., Zhang D., Wang C., Xing D. (2025). Airborne microbes: Sampling, detection, and inactivation. Crit. Rev. Biotechnol..

[51] Shivaram K.B., Bhatt P., Verma M.S., Clase K., Simsek H. (2023). Bacteriophage-based biosensors for detection of pathogenic microbes in wastewater. Sci. Total Environ..

[52] Papaioannou C., Geladakis G., Kommata V., Batargias C., Lagoumintzis G. (2023). Insights in Pharmaceutical Pollution: The Prospective Role of eDNA Metabarcoding. Toxics.

[53] Fronczek C.F., Yoon J.Y. (2015). Biosensors for Monitoring Airborne Pathogens. J. Lab. Autom..

[54] Marselle M.R., Hartig T., Cox D.T., De Bell S., Knapp S., Lindley S., Triguero-Mas M., Böhning-Gaese K., Braubach M., Cook P.A. (2021). Pathways linking biodiversity to human health: A conceptual framework. Environ. Int..

[55] Bass D., Christison K.W., Stentiford G.D., Cook L.S.J., Hartikainen H. (2023). Environmental DNA/RNA for pathogen and parasite detection, surveillance, and ecology. Trends Parasitol..

[56] Rishan S.T., Kline R.J., Rahman M.S. (2023). Applications of environmental DNA (eDNA) to detect subterranean and aquatic invasive species: A critical review on the challenges and limitations of eDNA metabarcoding. Environ. Adv..

[57] Vasavi S., Sripathi V., Simma C.M. (2024). Visualization of humpback whale tracking on edge device using space-borne remote sensing data for Indian Ocean. Egypt. J. Remote Sens. Space Sci..

[58] Sajjad B., Hussain S., Rasool K., Hassan M., Almomani F. (2023). Comprehensive insights into advances in ambient bioaerosols sampling, analysis and factors influencing bioaerosols composition. Environ. Pollut..

[59] Ruppert K.M., Kline R.J., Rahman M.S. (2019). Past, present, and future perspectives of environmental DNA (eDNA) metabarcoding: A systematic review in methods, monitoring, and applications of global eDNA. Glob. Ecol. Conserv..

[60] Yang J., Li C., Lo L.S.H., Zhang X., Chen Z., Gao J., Clara U., Dai Z., Nakaoka M., Yang H. (2024). Artificial Intelligence-Assisted Environmental DNA Metabarcoding and High-Resolution Underwater Optical Imaging for Noninvasive and Innovative Marine Environmental Monitoring. J. Mar. Sci. Eng..

[61] Valenzuela E.F., Menezes H.C., Cardeal Z.L. (2020). Passive and grab sampling methods to assess pesticide residues in water. A review. Environ. Chem. Lett..

[62] Pouresmaieli M., Ataei M., Forouzandeh P., Azizollahi P., Mahmoudifard M. (2022). Recent progress on sustainable phytoremediation of heavy metals from soil. J. Environ. Chem. Eng..

[63] Rasool S., Rasool T., Gani K.M. (2022). A review of interactions of pesticides within various interfaces of intrinsic and organic residue amended soil environment. Chem. Eng. J. Adv..

[64] Yahaya S.M., Mahmud A.A., Abdullahi M., Haruna A. (2023). Recent advances in the chemistry of nitrogen, phosphorus and potassium as fertilizers in soil: A review. Pedosphere.

[65] Zhao S., Yuan X.-T., Wang X.-H., Ai Y.-J., Li F.-P. (2024). Research Progress and Hotspots in Microbial Remediation for Polluted Soils. Sustainability.

[66] Abdelhamid M.A., Khalifa H.O., Yoon H.J., Ki M.-R., Pack S.P. (2024). Microbial Immobilized Enzyme Biocatalysts for Multipollutant Mitigation: Harnessing Nature’s Toolkit for Environmental Sustainability. Int. J. Mol. Sci..

[67] Abdelhamid M.A., Son R.G., Ki M.-R., Pack S.P. (2024). Biosilica-coated carbonic anhydrase displayed on Escherichia coli: A novel design approach for efficient and stable biocatalyst for CO2 sequestration. Int. J. Biol. Macromol..

[68] Ariza M., Fouks B., Mauvisseau Q., Halvorsen R., Alsos I.G., de Boer H.J. (2023). Plant biodiversity assessment through soil eDNA reflects temporal and local diversity. Methods Ecol. Evol..

[69] Hiiesalu I., Oepik M., Metsis M., Lilje L., Davison J., Vasar M., Moora M., Zobel M., Wilson S.D., Paertel M. (2012). Plant species richness belowground: Higher richness and new patterns revealed by next-generation sequencing. Mol. Ecol..

[70] Wendt J., Hauser S. (2013). An equivalent soil mass procedure for monitoring soil organic carbon in multiple soil layers. Eur. J. Soil Sci..

[71] Fu Y., Xue M., Cai R., Kangasluoma J., Jiang J. (2019). Theoretical and experimental analysis of the core sampling method: Reducing diffusional losses in aerosol sampling line. Aerosol. Sci. Technol..

[72] Hou D., O’Connor D., Nathanail P., Tian L., Ma Y. (2017). Integrated GIS and multivariate statistical analysis for regional scale assessment of heavy metal soil contamination: A critical review. Environ. Pollut..

[73] Young J.M., Rawlence N.J., Weyrich L.S., Cooper A. (2014). Limitations and recommendations for successful DNA extraction from forensic soil samples: A review. Sci. Justice.

[74] Roberts J., Cozzolino D. (2016). Wet or dry? The effect of sample characteristics on the determination of soil properties by near infrared spectroscopy. Trends Anal. Chem..

[75] Tuit C., Wait A. (2020). A review of marine sediment sampling methods. Environ. Forensics.

[76] He S., Peng Y., Jin Y., Wan B., Liu G. (2020). Review and analysis of key techniques in marine sediment sampling. Chin. J. Mech. Eng..

[77] Corinaldesi C., Barucca M., Luna G.M., Dell’anno A. (2011). Preservation, origin and genetic imprint of extracellular DNA in permanently anoxic deep-sea sediments. Mol. Ecol..

[78] Kestel J.H., Field D.L., Bateman P.W., White N.E., Allentoft M.E., Hopkins A.J., Gibberd M., Nevill P. (2022). Applications of environmental DNA (eDNA) in agricultural systems: Current uses, limitations and future prospects. Sci. Total Environ..

[79] Sorensen J.P., Maurice L., Edwards F.K., Lapworth D.J., Read D.S., Allen D., Butcher A.S., Newbold L.K., Townsend B.R., Williams P.J. (2013). Using boreholes as windows into groundwater ecosystems. PLoS ONE.

[80] Korbel K., Chariton A., Stephenson S., Greenfield P., Hose G.C. (2017). Wells provide a distorted view of life in the aquifer: Implications for sampling, monitoring and assessment of groundwater ecosystems. Sci. Rep..

[81] Britt S.L., Parker B.L., Cherry J.A. (2010). A downhole passive sampling system to avoid bias and error from groundwater sample handling. Environ. Sci. Technol..

[82] Harter T., Watanabe N., Li X., Atwill E.R., Samuels W. (2014). Microbial groundwater sampling protocol for fecal-rich environments. Groundwater.

[83] Gomo M., Vermeulen D., Lourens P. (2018). Groundwater sampling: Flow-through bailer passive method versus conventional purge method. Nat. Resour. Res..

[84] van der Heyde M., Alexander J., Nevill P., Austin A.D., Stevens N., Jones M., Guzik M.T. (2023). Rapid detection of subterranean fauna from passive sampling of groundwater eDNA. Environ. DNA.

[85] Couton M., Hürlemann S., Studer A., Alther R., Altermatt F. (2023). Groundwater environmental DNA metabarcoding reveals hidden diversity and reflects land-use and geology. Mol. Ecol..

[86] Wu P., Feng J., Ju M., Wu S., Han W., Wang M., Liao J., Zhao L., Gao Y., Zheng J. (2024). Water filter: A rapid water environmental DNA collector in the field. Front. Environ. Sci..

[87] Davis J., Garcia E.A., Gibb K.S., Kennard M.J., Rose A., Stromsoe N., Wedd D. (2023). The importance of groundwater for riverine fish faunas in a region of shale gas development in northern Australia. Front. Environ. Sci..

[88] Hossain M.S., Iken B., Iyer R. (2024). Whole genome analysis of 26 bacterial strains reveals aromatic and hydrocarbon degrading enzymes from diverse environmental soil samples. Sci. Rep..

[89] Ji L., Chang X., Wang L., Fu X., Lai W., Zheng L., Li Q., Xing Y., Yang Z., Guan Y. (2024). The Mechanism Insight into Bacterial Degradation of Pentachlorobiphenyl. bioRxiv.

[90] Chunyan X., Qaria M.A., Qi X., Daochen Z. (2023). The role of microorganisms in petroleum degradation: Current development and prospects. Sci. Total Environ..

[91] Guerrero Ramírez J.R., Ibarra Muñoz L.A., Balagurusamy N., Frías Ramírez J.E., Alfaro Hernández L., Carrillo Campos J. (2023). Microbiology and biochemistry of pesticides biodegradation. Int. J. Mol. Sci..

[92] Lemmel F., Maunoury-Danger F., Leyval C., Cébron A. (2021). Altered fungal communities in contaminated soils from French industrial brownfields. J. Hazard. Mater..

[93] Zhang R.-D., Gao F.-Z., Shi Y.-J., Zhao J.-L., Liu Y.-S., He L.-Y., Ying G.-G. (2024). Metagenomic investigation of antibiotic resistance genes and resistant bacteria contamination in pharmaceutical plant sites in China. Environ. Pollut..

[94] Mejia M.P., Rojas C.A., Curd E., Renshaw M.A., Edalati K., Shih B., Vincent N., Lin M., Nguyen P.H., Wayne R. (2023). Soil microbial community composition and tolerance to contaminants in an urban brownfield site. Microb. Ecol..

[95] Kavehei A., Hose G.C., Chariton A.A., Gore D.B. (2021). Application of environmental DNA for assessment of contamination downstream of a legacy base metal mine. J. Hazard. Mater..

[96] Alves Senabio J., Correia da Silva R., Guariz Pinheiro D., Gomes de Vasconcelos L., Soares M.A. (2024). The pesticides carbofuran and picloram alter the diversity and abundance of soil microbial communities. PLoS ONE.

[97] Xing K., Lu W., Huang Q., Wu J., Shang H., Wang Q., Guo F., Du Q., Yin Z., Zhang Y. (2024). Soil eDNA biomonitoring reveals changes in multitrophic biodiversity and ecological health of agroecosystems. Environ. Res..

[98] Brunetti M., Magoga G., Cussigh A., Alali S., Pizzi F., Cremonesi P., Di Lelio I., Becchimanzi A., Comolli R., Gallina P.M. (2024). Soil invertebrate biodiversity and functionality within the intensively farmed areas of the Po Valley. Appl. Soil Ecol..

[99] Ruppert O.M., Homola J.J., Kanefsky J., Swinehart A., Scribner K.T., Robinson J.D. (2025). Optimization of Wetland Environmental DNA Metabarcoding Protocols for Great Lakes Region Herpetofauna. Environ. DNA.

[100] Angeles I.B., Romero-Martínez M.L., Cavaliere M., Varrella S., Francescangeli F., Piredda R., Mazzocchi M.G., Montresor M., Schirone A., Delbono I. (2023). Encapsulated in sediments: eDNA deciphers the ecosystem history of one of the most polluted European marine sites. Environ. Int..

[101] Kavehei A., Gore D.B., Chariton A.A., Hose G.C. (2021). Impact assessment of ephemeral discharge of contamination downstream of two legacy base metal mines using environmental DNA. J. Hazard. Mater..

[102] Agerbo Rasmussen J., Nielsen M., Mak S.S., Döring J., Klincke F., Gopalakrishnan S., Dunn R.R., Kauer R., Gilbert M.T.P. (2021). eDNA-based biomonitoring at an experimental German vineyard to characterize how management regimes shape ecosystem diversity. Environ. DNA.

[103] Frøslev T.G., Nielsen I.B., Santos S.S., Barnes C.J., Bruun H.H., Ejrnæs R. (2022). The biodiversity effect of reduced tillage on soil microbiota. Ambio.

[104] Le Provost G., Thiele J., Westphal C., Penone C., Allan E., Neyret M., Van Der Plas F., Ayasse M., Bardgett R.D., Birkhofer K. (2021). Contrasting responses of above-and belowground diversity to multiple components of land-use intensity. Nat. Commun..

[105] Shackleton M., Rees G.N., Watson G., Campbell C., Nielsen D. (2019). Environmental DNA reveals landscape mosaic of wetland plant communities. Glob. Ecol. Conserv..

[106] Tetzlaff S.J., Katz A.D., Wolff P.J., Kleitch M.E. (2024). Comparison of soil eDNA to camera traps for assessing mammal and bird community composition and site use. Ecol. Evol..

[107] Randall L.A., Goldberg C.S., Moehenschlager A. (2023). Environmental DNA surveys can underestimate amphibian occupancy and overestimate detection probability: Implications for practice. J. Wildl. Manag..

[108] Xie G., Lan J., Liang J., Wang Q., Cao X., Wang Y., Ren C., Liu H., Zhang J. (2024). Biodiversity and distribution of zoobenthos in the ecological water replenishment area of the Yellow River estuary coastal wetland revealed by eDNA metabarcoding. PLoS ONE.

[109] Pedreira-Segade U., Hao J., Razafitianamaharavo A., Pelletier M., Marry V., Le Crom S., Michot L.J., Daniel I. (2018). How do nucleotides adsorb onto clays?. Life.

[110] Abdelhamid M.A., Ki M.-R., Pack S.P. (2024). Biominerals and Bioinspired materials in Biosensing: Recent advancements and applications. Int. J. Mol. Sci..

[111] Tedetti M., Sempéré R. (2006). Penetration of ultraviolet radiation in the marine environment. A review. Photochem. Photobiol..

[112] Guthrie A.M., Cooper C.E., Bateman P.W., van der Heyde M., Allentoft M.E., Nevill P. (2024). A quantitative analysis of vertebrate environmental DNA degradation in soil in response to time, UV light, and temperature. Environ. DNA.

[113] Sirois S.H., Buckley D.H. (2019). Factors governing extracellular DNA degradation dynamics in soil. Environ. Microbiol. Rep..

[114] Barnes M.A., Turner C.R., Jerde C.L., Renshaw M.A., Chadderton W.L., Lodge D.M. (2014). Environmental conditions influence eDNA persistence in aquatic systems. Environ. Sci. Technol..

[115] Rees H.C., Maddison B.C., Middleitch D.J., Patmore J.R.M., Gough K.C. (2014). REVIEW: The detection of aquatic animal species using environmental DNA—a review of eDNA as a survey tool in ecology. J. Appl. Ecol..

[116] Farrell J.A., Whitmore L., Duffy D.J. (2021). The promise and pitfalls of environmental DNA and RNA approaches for the monitoring of human and animal pathogens from aquatic sources. BioScience.

[117] Paruch A.M., Paruch L. (2024). Current status of microbial source tracking applications in constructed wetlands serving as nature-based solutions for water management and wastewater treatment. Environ. Pollut..

[118] Sivalingam P., Sabatino R., Sbaffi T., Corno G., Fontaneto D., Borgomaneiro G., Rogora M., Crotti E., Mapelli F., Borin S. (2024). Cesare, A.D. Anthropogenic pollution may enhance natural transformation in water, favouring the spread of antibiotic resistance genes. J. Hazard. Mater..

[119] Wang B., Wang Y., He N., Du M., You P. (2024). Exploring riverine aquatic animal diversity and establishing aquatic ecological monitoring approaches tailored to the Qinling region via eDNA technology. Intergr. Zool..

[120] Suren A.M., Burdon F.J., Wilkinson S.P. (2024). eDNA is a useful environmental monitoring tool for assessing stream ecological health. Environ. DNA.

[121] Cornman R.S., Mckenna J.E., Fike J., Oyler-McCance S.J., Johnson R. (2018). An experimental comparison of composite and grab sampling of stream water for metagenetic analysis of environmental DNA. PeerJ.

[122] Schwesig K., Zizka V., Scherver C., Holzel N. (2024). Comparing eDNA and transect methos for aquatic biodiversity assessment in lakes and ponds. Mol. Ecol. Resour..

[123] Govindarajan A.F., McCartin L., Adams A., Allan E., Belani A., Francoline R., Fujii J., Gomez-Ibanez D., Kukulya A., Marin F. (2022). Improved biodiversity detection using a large-volume environmental DNA sampler with in situ filtration and implications for marine eDNA sampling strategies. Deep. Sea Res. Part I Oceanogr. Res. Pap..

[124] Tadic D., Manasfi R., Bertrand M., Sauvetre A., Chiron S. (2022). Use of passive and grab sampling and high-resolution mass spectrometry for non-targeted analysis of emerging contaminants and their semi-quantification in water. Molecules.

[125] Kotlash A.R., Chessman B.C. (1998). Effects of water sample preservation and storage on nitrogen and phosphorus determinations: Implications for the use of automated sampling equipment. Water. Res..

[126] Coes A.L., Paretti N.V., Foreman W.T., Iverson J., Alvarez D.A. (2014). Sampling trace organic compounds in water: A comparison of a continuous active sampler to continuous passive and discrete sampling methods. Sci. Total Environ..

[127] Kot A., Zabiegała B., Namieśnik J. (2000). Passive sampling for long-term monitoring of organic pollutants in water. Trac-Trends Anal. Chem..

[128] Chen C., Zhang H., Jones K.C. (2012). A novel passive water sampler for in situ sampling of antibiotics. J. Environ. Monit..

[129] Chen C., Zhang H., Ying G.G., Jones K.C. (2013). Evidence and recommendations to support the use of a novel passive water sampler to quantify antibiotics in wastewaters. Environ. Sci. Technol..

[130] Schwarzbach M., Laiacker M., Pazmany M.M., Kondak K. Remote water sampling using flying robots. Proceedings of the 2014 International Conference on Unmanned Aircraft Systems (ICUAS).

[131] Boger N., Ozer M. (2023). Monitoring sewer systems to detect the eDNA of missing persons and persons of interest. Forensic Sci. Int..

[132] Choi P.M., Tscharke B.J., Donner E., O’Brien J.W., Grant S.C., Kaserzon S.L., Mackie R., O’Malley E., Crosible N.D., Thomas K.V. (2018). Wastewater-based epidemiology biomarkers: Past, present, and future. Trac-Trends Anal. Chem..

[133] Nguyen A.Q., Vu H.P., Nguyen L.N., Wang Q., Djordjevic S.P., Donner E., Yin H., Nghiem L.D. (2021). Monitoring antibiotic resistance genes in wastewater treatment: Current strategies and future challenges. Sci. Total Environ..

[134] Hata A., Honda R. (2020). Potential sensitivity of wastewater monitoring for SARS-CoV-2: Comparison with norovirus cases. Environ. Sci. Technol..

[135] Lajoie A.S., Holm R.H., Anderson L.B., Ness H.D., Smith T. (2022). Nationwide public perceptions regarding the acceptance of using wastewater for community health monitoring in the United States. PLoS ONE.

[136] Osunmakinde C.O., Selvarajan R., Mamba B.B., Msagati A.M. (2019). Profiling bacterial diversity and potential pathogens in wastewater treatment plats using high throughput sequencing analysis. Microorganisms.

[137] Oladi M., Leontidou K., Stoeck T., Shokri M.R. (2022). Environmental DNA-based profiling of benthic bacterial and eukaryote communities along a crude oil spill gradient in a coral reef in the Persian Gulf. Mar. Pollut. Bull..

[138] Ki M.-R., Kim S.H., Park T.I., Pack S.P. (2023). Self-entrapment of antimicrobial peptides in silica particles for stable and effective antimicrobial peptide delivery system. Int. J. Mol. Sci..

[139] Park K.S., Choi A., Park T.-I., Pack S.P. (2024). Fluorometric and Colorimetric Method for SARS-CoV-2 Detection Using Designed Aptamer Display Particles. Biosensors.

[140] Min K.H., Kim K.H., Ki M.-R., Pack S.P. (2024). Antimicrobial peptides and their biomedical applications: A review. Antibiotics.

[141] Rishan S.T., Kline R.J., Rahman M.S. (2024). Exploitation of environmental DNA (eDNA) for ecotoxicological research: A critical review on eDNA metabarcoding in assessing marine pollution. Chemosphere.

[142] Hernandez-Alomia F., Ballesteros I., Castillejo P. (2022). Bioremediation potential of glyphosate-degrading microorganisms in eutrophicated Ecuadorian water bodies. Saudi J. Biol. Sci..

[143] Song T., Zi F., Huang Y., Fang L., Zhang Y., Liu Y., Chang J., Li J. (2025). Assessment of aquatic ecosystem health in the Irtysh river basin using eDNA metabarcoding. Water.

[144] Levy N., Simon-Blecher N., Ben-Ezra S., Yuval M., Doniger T., Leray M., Karako-Lampert S., Tarazi E., Levy O. (2023). Evaluating biodiversity for coral reef reformation and monitoring on complex 3D structures using environmental DNA (eDNA) metabarcoding. Sci. Total Environ..

[145] Manaff A.H.N.A., Hil K.S., Luo Z., Liu M., Law I.K., Teng S.T., Akhir M.F., Gu H., Leaw C.P., Lim P.T. (2023). Mapping harmful microalgal species by eDNA monitoring: A large-scale survey across the southwestern South China Sea. Harmful Algae.

[146] Johnson G., Nour A.A., Nolan T., Huggett J., Bustin S. (2014). Minimum information necessary for quantitative real-time PCR experiments. Quantitative Real-Time PCR: Methods and Protocols.

[147] Cunningham S.W., Tessler M., Johnson-Rosemond J., Whittaker I.S., Brugler M.R. (2024). Environmental DNA Isolation, Validation, and Preservation Methods. DNA Barcoding: Methods and Protocols.

[148] Bruno F., Marinella M., Santamaria M. (2014). e-DNA meta-barcoding: From NGS raw data to taxonomic profiling. RNA Bioinformatics.

[149] Christensen H., Olsen J.E. (2023). Full Shotgun DNA Metagenomics. Introduction to Bioinformatics in Microbiology.

[150] Wood S.A., Pochon X., Laroche O., von Ammon U., Adamson J., Zaiko A. (2019). A comparison of droplet digital polymerase chain reaction (PCR), quantitative PCR and metabarcoding for species-specific detection in environmental DNA. Mol. Ecol. Resour..

[151] Sruoga V., Stunzenas V., Paulaviciute B. (2009). COI Gene as a Molecular Marker of Elachista Species (Lepidoptera: Elachistidae: Elachistinae) from Different Lithuanian Populations.

[152] Panicker V.P., Haridas P.C., Narayanan A., Mohammed S., Babu B.C. (2019). Mitochondrial 12S rRNA gene sequence analysis, a tool for species identification. J. Wildl. Biodivers..

[153] Kõljalg U., Nilsson R.H., Abarenkov K., Tedersoo L., Taylor A.F., Bahram M., Bates S.T., Bruns T.D., Bengtsson-Palme J., Callaghan T.M. (2013). Towards a unified paradigm for sequence-based identification of fungi. Mol. Ecol..

[154] Wang P., Yan Z., Yang S., Wang S., Zheng X., Fan J., Zhang T. (2019). Environmental DNA: An emerging tool in ecological assessment. Bull. Environ. Contam. Toxicol..

[155] Deiner K., Bik H.M., Mächler E., Seymour M., Lacoursière-Roussel A., Altermatt F., Creer S., Bista I., Lodge D.M., De Vere N. (2017). Environmental DNA metabarcoding: Transforming how we survey animal and plant communities. Mol. Ecol..

[156] Olawade D.B., Wada O.Z., Ige A.O., Egbewole B.I., Olojo A., Oladapo B.I. (2024). Artificial intelligence in environmental monitoring: Advancements, challenges, and future directions. Hyg. Environ. Health Adv..

[157] Ahuja A., Al-Zogbi L., Krieger A. (2021). Application of noise-reduction techniques to machine learning algorithms for breast cancer tumor identification. Comput. Biol. Med..

[158] Eraslan G., Avsec Ž., Gagneur J., Theis F.J. (2019). Deep learning: New computational modelling techniques for genomics. Nat. Rev. Genet..

[159] Frontalini F., Greco M., Semprucci F., Cermakova K., Merzi T., Pawlowski J. (2025). Developing and testing a new Ecological Quality Status index based on marine nematode metabarcoding: A proof of concept. Chemosphere.

[160] Whitmore L., McCauley M., Farrell J.A., Stammnitz M.R., Koda S.A., Mashkour N., Summers V., Osborne T., Whilde J., Duffy D.J. (2023). Inadvertent human genomic bycatch and intentional capture raise beneficial applications and ethical concerns with environmental DNA. Nat. Ecol. Evol..

